# Case report: aseptic meningitis secondary to varicella-zoster virus (VZV) without an exanthem post MMR vaccination

**DOI:** 10.1186/s12879-021-06453-5

**Published:** 2021-08-03

**Authors:** Syeda Sahra, Abdullah Jahangir, Allison Glaser, Neville Mobarakai, Ahmad Jahangir

**Affiliations:** 1 Department of Internal Medicine, Hofstra School of Medicine, Staten Island University Hospital, 475-Seaview Avenue, Staten Island, NY 10305 USA; 2 King Edward Medical University, Lahore, 10305 Pakistan

**Keywords:** Aseptic meningitis, Chickenpox, MMR, Shingles, VZV, Zoster

## Abstract

**Background:**

Varicella-zoster virus (VZV) is a known cause of aseptic meningitis, with a predisposition for an immunocompromised population. A dermatomal rash usually accompanies aseptic meningitis secondary to VZV.

**Case presentation:**

We report the case of a 31-year-old male with a history of chickenpox in childhood and recent shingles who presented with severe frontal headaches secondary to VZV meningitis. The patient had also recently received the measles-mumps-rubella (MMR) vaccine. He recovered without any neurological sequala.

**Conclusion:**

This case report describes an immunocompetent patient with recent MMR vaccination who developed aseptic meningitis secondary to VZV without any dermatomal involvement (Zoster Sine Herpete).

## Background

Varicella-zoster virus (VZV) is an alphaherpesvirus with a broad range of clinical presentations, including chickenpox in early childhood and herpes zoster (shingles) later in life. Pathogenesis of shingles involves reactivation of virus from the dormant phase in neuronal ganglia. Various triggers, including immunocompromised status, lipid-lowering statins, and psychological stress, can lead to VZV reactivation. VZV infection can lead to aseptic meningitis in both immunocompetent and immunocompromised patients. VZV can also present without a dermatomal rash (Zoster Sine Herpete). We present the case of a young male who presented with headaches and was diagnosed with aseptic meningitis secondary to VZV reactivation without any dermatomal involvement. We ruled out other viral causes of aseptic meningitis based on CSF PCR studies. The only possible risk factor was a recent MMR (measles, mumps, rubella) vaccination. MMR vaccination was notoriously associated with outbreaks of aseptic meningitis in the past after mass vaccination programs [1–8]. This case is presented to consider VZV as a possible cause of viral meningitis in immunocompetent patients with Zoster Sine Herpete in the backdrop of recent MMR vaccination.

## Case presentation

A 31-year-old-male with no former medical and surgical history presented to the emergency room (ER) with an acute headache for 5 days in the summer of 2020. The patient noticed symptoms initially while lifting weights at the gymnasium. The headache was localized frontally. The pain was described as sharp and non-pulsating in nature and 10/10 in intensity. It worsened gradually and was associated with nausea and one episode of non-bilious, non-bloody vomiting. He took over-the-counter NSAIDs for the headaches with no improvement.

He denied any recent trauma, sick contacts, viral illness, blurred vision, diplopia, photophobia, tinnitus, hearing loss, neck stiffness, ataxia, focal motor or sensory, neurological deficits, cough, fever, chills, abdominal or chest pain, alteration in bowel habits, personal or family history of stroke and autoimmune diseases. The patient has been healthy and took no medications. He was up to date with his vaccinations and received his MMR vaccine the prior week as part of his workplace requirement. The patient was USA-based and was confirmed to have received the MMR vaccine manufactured by Merck Pharmaceuticals. He reported two dermatomal varicella-zoster virus infection episodes in the past, first during childhood and second at 29 years. There were no other complications, and the recovery was uneventful.

**Fig. 1:**
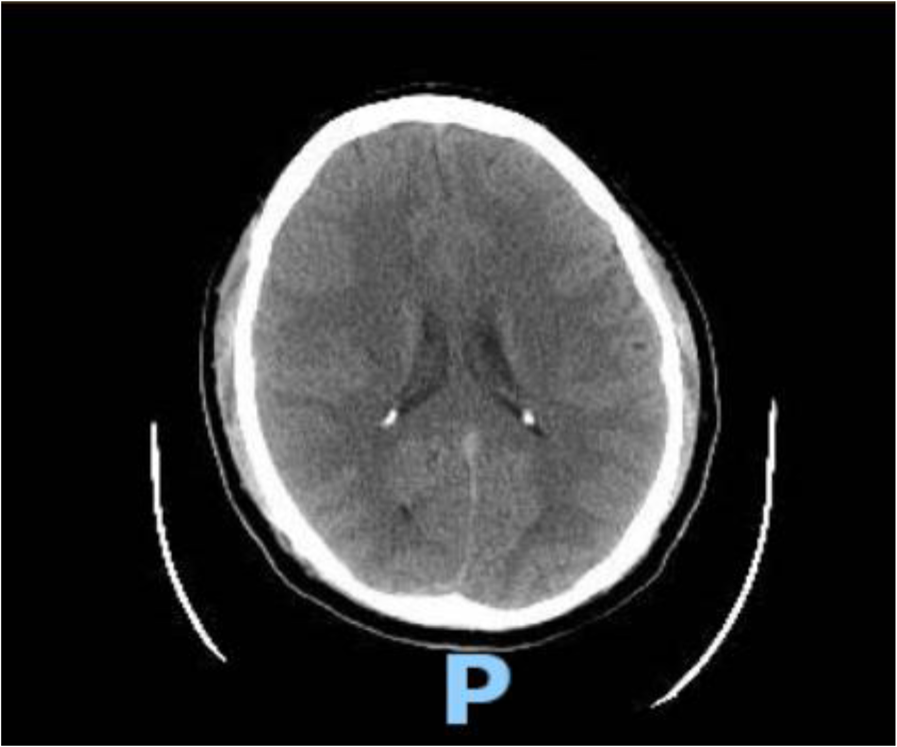
CT head with unremarkable findings (Axial plane)

**Fig. 2:**
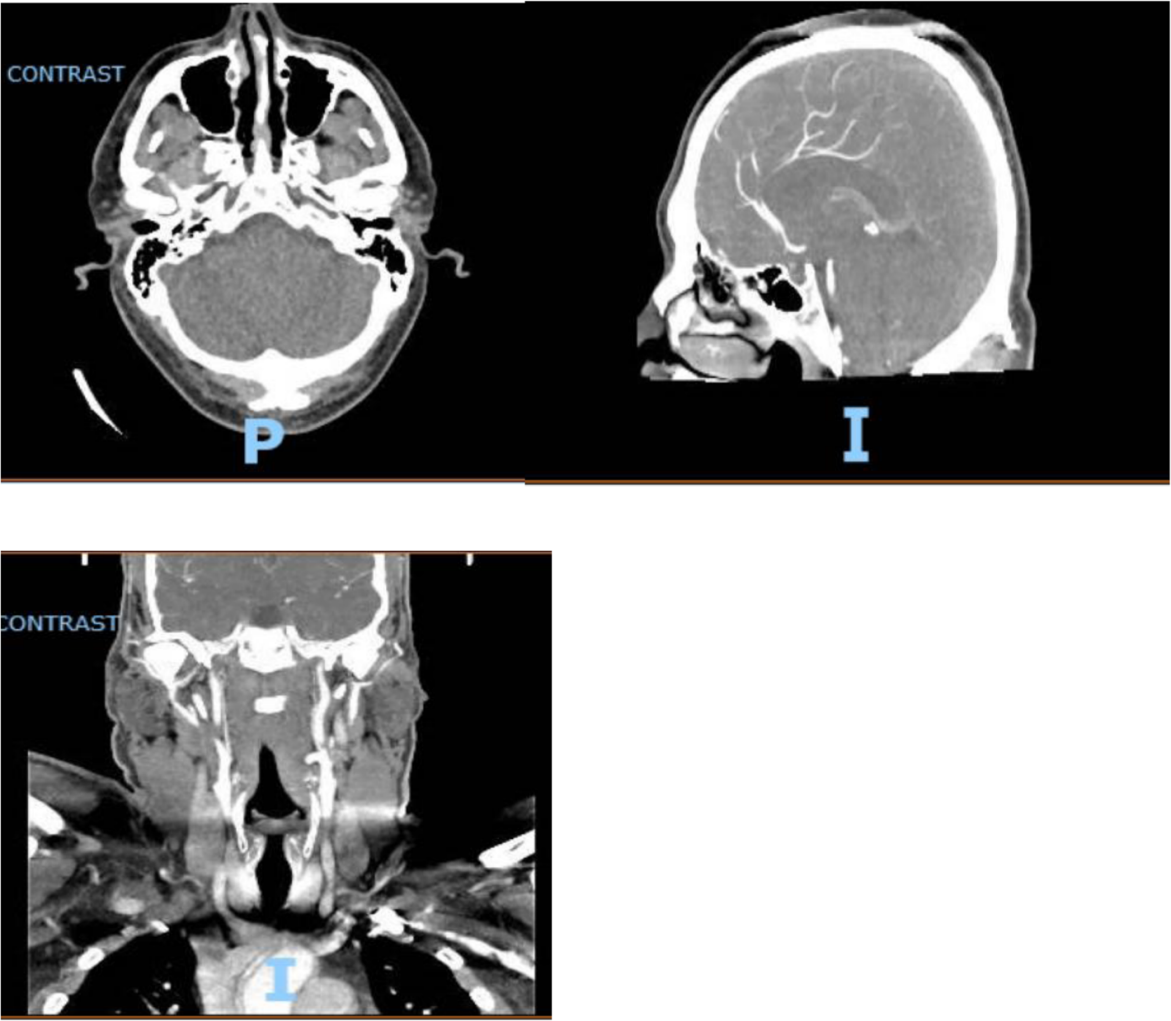
CT angiogram with IV contrast head and neck with patent circulation. **A** Axial (transverse) plane of head. **B** Sagittal plane of head. **C** Coronal plane of head

On physical examination, he appeared uncomfortable and complained of a persistent frontal headache. Vitals were within normal limits, and physical examination was unremarkable without any dermatomal rash. Signs of meningeal irritation were negative. COVID-19 PCR and COVID-19 antibody testing were negative. CT head and CT angiogram head and neck were unremarkable (Figs. 1 and 2).

Lumbar puncture was performed, which showed elevated proteins (86 mg/dL) and mononuclear cells with a lymphocytic predominance (96%) on initial studies suggestive of viral meningitis (Table 1).

**Table 1:** Initial CSF studies

CSF Findings	Tube 1	Tube 2
CSF Color	No color	No color
Appearance spun	Colorless	Colorless
CSF monocytes/macrophages (15–45%)	4%	8%
CSF segmented neutrophils (0–6%)	0%	1%
Total nucleated cells, CSF (0–5 u/L)	460 u/L	630 u/L
CSF appearance	Clear	Clear
CSF lymphocytes (40–80%)	96%	91%
RBC count spinal fluid (0–0 u/L)	6	0
LDH, CSF	28 U/L	
Protein, CSF (15-45 mg/dL)	86	
Glucose, CSF (45–75 mg/dL)	56	

The patient received empiric overage of IV acyclovir (10 mg/kg every 8 h) and ceftriaxone (2 g every 12 h) based on cytology of lumbar puncture studies. The patient reported an improvement of his symptoms on hospital day 2 with an appropriate pain control regimen. CSF PCR studies (performed on tube 2) were positive for VZV PCR (6800 copies/mL) and negative for Herpes Simplex Virus 1 and 2 (Table 2). Diagnosis of aseptic meningitis secondary to VZV was made. VZV IgG came back positive. Intravenous acyclovir with appropriate hydration was continued. The patient’s headache resolved gradually, and he was discharged home (hospital day 4) on oral acyclovir for 10 days. The patient was followed up after discharge and was doing well with complete resolution of symptoms.

**Table 2:** CSF PCR studies

CSF PCR Result	Positive for VZV
VZV DNA PCR	68,000 copies/mL
COVID-19 IgG Antibody Index	Negative
Varicella IgG antibody (Index)	2073.0
Varicella IgG interpretation	Positive
Varicella-Zoster IgM (0.00–0.90)	< 0.91 (borderline)
West Nile IgM	Negative
West Nile IgG	Negative
West Nile Virus by PCR	Not detected
CSF cultures	No growth
HSV ½ PCR	Negative
*E. coli* K1	Negative
*Streptococcus agalactiae*	Negative
*Streptococcus pneumoniae*	Negative
Cytomegalovirus	Negative
Human Herpes Virus 6	Negative
Human parechovirus	Negative
*Cryptococcus neoformans/gattii*	Negative
Lyme PCR, Result	Negative

## Discussion and conclusion

Varicella-Zoster Virus (VZV) is well known for causing chickenpox (varicella) in children and shingles (zoster) in the elderly, first described in 1958 [9]. The transmission is through contact and direct inhalation of aerosols from infected secretions. The reactivation of dormant VZV from spinal ganglia from a prior infection is attributed to zoster [10]. Psychological stress, concurrent co-morbidities like uncontrolled diabetes, and the use of statins (cholesterol-lowering drugs) have been associated with the possible reactivation of VZV. The immune arm responsible specifically for VZV (memory T cells) can decline after some time and contribute to the viral renaissance. This phenomenon can explain the higher incidence of shingles with advanced age [11]. Genomic studies are underway to understand the mechanism of reactivation as it is a widespread phenomenon for at least one-third of the population infected with VZV [12]. The reactivation leads to shingles which are followed by painful and debilitating postherpetic neuralgia for months. In Zoster Sine Herpete, the viral reactivation occurs at the neuronal level, but the viral migration to cutaneous tissue is absent.

VZV is an established cause of aseptic meningitis with a range of neurological manifestations even in immunocompetent populations, more so in men [13–18]. The pure VZV virus isolation is cumbersome, and diagnosis is made based on positive VZV DNA PCR and Antibodies [19]. Our [4, 12, 13, 20] institutional assay range for VZV PCR is set at 252 copies/mL to 1.00E+ 08 copies/mL. Viracor Eurofins developed this test and its performance characteristics. The VZV IgG antibody was positive, performed by Chemiluminescence Immunoassay with high index (2073), where < 135 indexes is considered negative. This test is negative in infected patients during the incubation period and early stages of infection. Index 135–16 5 is equivocal. Age group appropriate vaccination schedules are available worldwide to avoid the VZV complications of chickenpox and zoster [21]. Acyclovir is the treatment of choice, supplemented with aggressive hydration to avoid kidney injury.

This case is unusual as the patient was immunocompetent and developed aseptic meningitis without any exanthem or neuralgia. He had developed chickenpox in his childhood and shingles 2 years prior. We tested the patient for the Human immunodeficiency virus (HIV), which came back negative. COVID-19 PCR and antibody testing were negative as well. No underlying co-morbidity or immunosuppressive state could be attributed to repeated infection with VZV. A case of aseptic meningitis secondary to VZV with a similar clinical presentation recently was reported in the literature, but that particular patient had developed zoster rash without preherpetic or postherpetic neuralgia [22].

Our patient had received MMR recently, and cases of aseptic meningitis (not specifically VZV meningitis) have been reported after recent vaccinations, especially with MMR vaccine covering Urabe strain with mass outbreaks in different parts of the world [1–8]. The vaccines introduced later were associated with fewer neurological adverse events. This patient, as mentioned earlier, had received the MMR vaccine by Merck pharmaceuticals in the USA. He presented with a complaint of headache alone without any signs of meningeal irritation. The timeline for developing aseptic meningitis after vaccination is noticeably short in this patient. This fact might suggest the association to be of chance alone. Regardless, the suspicion of aseptic meningitis should arise with vague symptoms in the setting of recent vaccination. Mamishi et al. (2016) and Sugiura et al. (1991) studied the incidence of aseptic meningitis after MMR vaccination. Their studies indicated that the male gender and younger age group were associated with an increased incidence of aseptic meningitis after vaccination [4, 20]. Similar studies in adolescent and elderly age groups have not been done as mass vaccinations target the younger populations as part of their routine vaccinations.

The case is being reported to keep VZV meningitis in differential diagnoses in patients presenting with signs and CSF findings suggestive of viral meningitis without any dermatomal rash or immunocompromising medical conditions history of recent vaccination. We can study the period since recent vaccination or viral illness in cases with similar clinical presentations for future monitoring purposes. The vaccine batch can also be traced for microbiological analysis if analogous cases are reported in the future in succession.

## Data Availability

The datasets used or analyzed during the current case reports are available from the corresponding author on reasonable request.

## Abbreviations

MMR: Measles-mumps-rubella
VZV: Varicella zoster virus
CFS: Cerebrospinal fluid
PCR: Polymerase chain reaction
